# Supplementary figures and images for: Chemo- and optogenetic activation of hypothalamic Foxb1-expressing neurons and their terminal endings in the rostral-dorsolateral PAG leads to tachypnea, bradycardia, and immobility
Source: eLife. 2024 Feb 1;12:RP86737. doi: 10.7554/eLife.86737 (PMC10945554; doi:10.7554/eLife.86737)

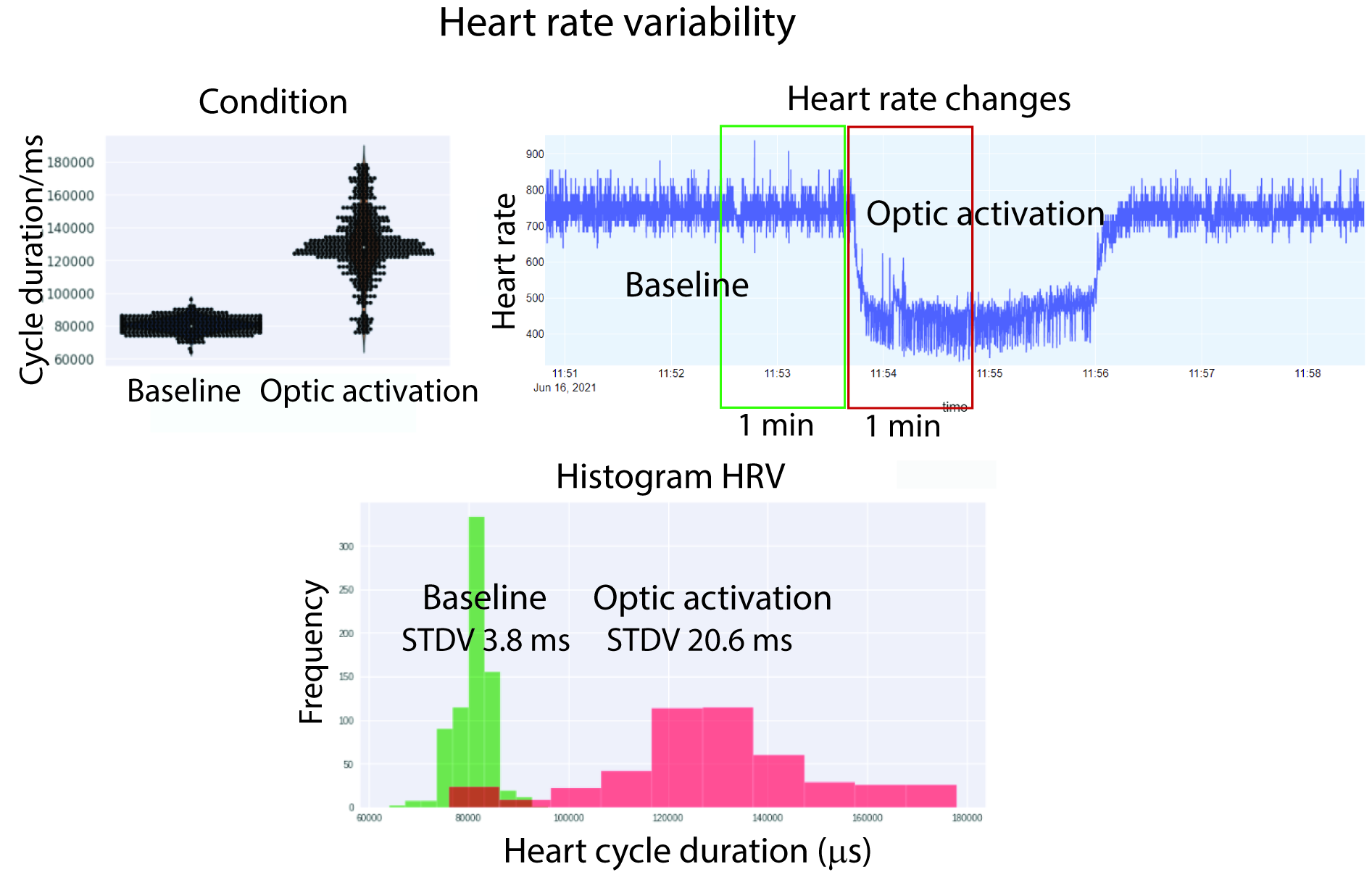

Supplement: Supplementary file 5. — Comparison between the one-minute period before (green rectangle) and after (red rectangle) the start of the optogenetic activation. While all 4 parameters changed significantly during optogenetic stimulation (heart rate, systolic BP, diastolic BP, mean BP), the SD of heart cycle duration changed by a factor of more than five, from 3.8ms during the baseline period to 20.6ms during optogenetic activation. [file elife-86737-supp5.tif]
